# Severe Drug-Induced Liver Injury From Over-the-Counter Medication

**DOI:** 10.7759/cureus.33558

**Published:** 2023-01-09

**Authors:** Kasumi Satoh, Manabu Okuyama, Nobuhisa Hirasawa, Hajime Nakae

**Affiliations:** 1 Advanced Emergency and Critical Care Center, Akita University Hospital, Akita, JPN

**Keywords:** drug-induced liver injury, liver injury, herbal medicines, kampo medicine (japanese herbal medicine), liver dysfunction, intensive care unit

## Abstract

Drug-induced liver injury (DILI) is difficult to diagnose as it presents with a wide variety of clinical manifestations and there is no established specific biomarker. However, clinicians require expertise in diagnosing DILI as it can lead to critical illness, is relatively common, and can be caused by a variety of drugs, herbal medicines, and supplements. A 67-year-old male was admitted to the hospital with a fever, jaundice, and fatigue. Abdominal ultrasonography, computed tomography, and magnetic resonance cholangiopancreatography revealed no morphological abnormalities in the hepatobiliary system. On the third day of hospitalization, the liver damage and acute kidney injury progressed, and the patient was transferred to our intensive care unit. To further investigate the cause of multiple organ damage, the patient underwent repeated history taking and additional laboratory testing. In addition to the common causes of hepatic and renal damage, we also tested for rickettsiosis and leptospirosis, as the patient reported partaking regularly in outdoor leisure activities. On day seven of hospitalization, the patient recalled taking over-the-counter herbal flu medications approximately five days prior to admission; therefore, we suspected DILI and performed a drug-induced lymphocyte stimulation test (DLST). The DLST was positive for one drug. As other causes had been ruled out, the patient was diagnosed with severe DILI. The clinical course of the patient was observed with the patient’s laboratory data and fever improving spontaneously. This case taught us several important lessons for the investigation of liver injury. Firstly, even with over-the-counter drugs, liver injury can be severe. Secondly, while the DLST is available for investigating DILI, false positives, especially for medicinal herbs, should be noted, and it is necessary to adequately rule out other diseases. Finally, when the cause of liver injury is unclear, patient history taking should be repeated carefully.

## Introduction

Drug-induced liver injury (DILI) is difficult to diagnose as it presents with a wide variety of clinical manifestations and there is no established specific biomarker [1]. However, clinicians require expertise in diagnosing DILI as it can lead to critical illness, is relatively common, and can be caused by a variety of drugs, herbal medicines, and supplements.

Patient history taking is of utmost importance. Engel and Morgan described the medical interview as "the most powerful and sensitive and most versatile instrument available to the physician,” and this is still true today, after half a century [2]. Moreover, the significance of patient medical history is remarkable, having been reported to lead to a final diagnosis in 76% of internal medicine patients [3].

In this report, we describe a case of severe drug-induced organ damage caused by an over-the-counter (OTC) herbal drug. This was initially managed as a liver disorder of an undetermined cause; however, after repeated patient history taking, a hidden etiology was revealed.

## Case presentation

**Figure 1:**
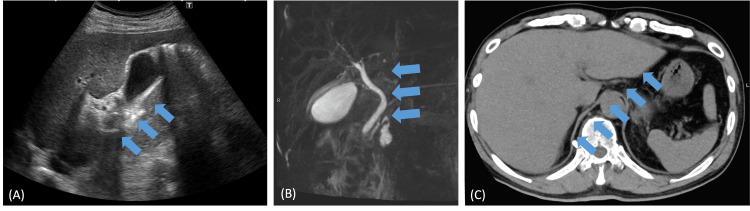
Imaging studies of the hepatobiliary system (A) Ultrasonography, (B) magnetic resonance cholangiopancreatography, and (C) non-enhanced computed tomography. No morphological abnormality was found in the hepatobiliary system (arrows).

A 67-year-old male was admitted to the hospital with a fever, jaundice, and fatigue. He had hypertension and was taking telmisartan (40 mg) and amlodipine besylate (5 mg) for the long term. He had no history of allergies, drank approximately 80 grams of alcohol equivalent daily, had a desk job, and played baseball on his days off. He had not recently eaten shellfish or wild game meat and had not ingested any toxic plants. Abdominal ultrasonography, computed tomography (CT), and magnetic resonance cholangiopancreatography (MRCP) were performed to investigate the cause of jaundice; however, no morphological abnormalities were observed in the hepatobiliary system (Figure 1). As such, he was diagnosed with a bacterial infection due to organ damage with systemic inflammatory response syndrome (SIRS). He was treated with cefotiam 1 g twice daily. However, on the third day of hospitalization, his liver damage and acute kidney injury progressed, and the patient was transferred to our hospital for intensive care.

During the physical examination, his blood pressure, pulse rate, respiratory rate, oxygen saturation, and body temperature were 115/55 mmHg with little norepinephrine (maximum: 0.08 μg/kg/min), 120 beats/minute with atrial fibrillation, 30 breaths/minute, 98%, and 38.0°C, respectively. A small dose of norepinephrine was required; however, there was no prolonged shock. There were no specific physical findings in the abdomen; however, he had marked jaundice all over his body and localized urticaria on the right anterior chest.

The laboratory tests indicated prominent cholestatic liver damage, kidney injury, and inflammatory findings. Meanwhile, the patient’s coagulation ability was almost maintained (Table 1).

**Table 1:** Laboratory test results on admission to our intensive care unit

Laboratory tests	Results	Normal range
White blood cell counts (/μL)	14,300	3,900-9,800
Hemoglobin (g/dL)	12.3	13.5-17.6
Platelet count (×10^3^/μL)	205	131-362
Aspartate aminotransferase (U/L)	32	10-40
Alanine aminotransferase (U/L)	45	5-40
γ-glutamyl transpeptidase (U/L)	372	<70
Total bilirubin (mg/dL)	13.6	0.3-1.2
Direct bilirubin (mg/dL)	12.6	<0.4
Alkaline phosphatase	427	38-113
Ammonia (μg/dL)	35	30-80
Amylase (mg/dL)	366	37-125
Lipase (mg/dL)	733	13-55
Creatinine (mg/dL)	2.44	0.17-1.00
Blood urea nitrogen (mg/dL)	47.3	8.0-22.0
C-reactive protein (U/L)	24.6	<0.14
Activated partial thromboplastin time (sec)	27.2	24.3-36.0
Prothrombin time (% in the normal range)	96.0	70-130
Fibrin/fibrinogen degradation products (μg/mL)	17.8	<4.0
D-dimer (μg/mL)	6.08	<1.0
Fibrinogen (mg/dL)	932	150-400

Although the patient was in SIRS, there was no infection focus on imaging, and both blood and urine cultures were negative. Moreover, the clinical response to antibiotics was unfavorable; therefore, they were promptly discontinued assuming that organ damage due to sepsis was unlikely. To further investigate the cause of the multiple organ damage, the patient and his family underwent repeated history taking and additional laboratory testing, as shown in Table 2. In addition to the common causes of hepatic and renal damage, we tested for rickettsiosis and leptospirosis as they reported partaking in outdoor leisure activities regularly.

**Table 2:** Results of the investigation for the cause of liver and kidney impairment Unless otherwise specified, serological tests were all taken during the acute phase. * Taken before antibiotic administration. IG, immunoglobulin; PCR, polymerase chain reaction; HA, hepatitis A; HB, hepatitis B; HCV, hepatitis C virus; HEV, hepatitis E virus; VCA, virus capsid antigen; EA, early antigen; EBNA, Epstein-Barr nuclear antigen; GBM, glomerular basement membrane; PR-3 ANCA, proteinase-3-antineutrophil cytoplasmic antibody; MPO-ANCA, myeloperoxidase antineutrophil cytoplasmic antibody.

Laboratory tests	Results
Bacteriological investigation	
Blood culture	Negative*
Urine culture	Negative*
Endotoxin	Negative
Mycoplasma pneumoniae, serological tests	Particle agglutination method: negative, complement fixation method: negative
Orientia tsutsugamushi	Serological test: IgG (-), IgM (-) PCR: negative
Rickettsia japonica	Serological test: IgG (-), IgM (-) PCR: negative
Leptospirosis (15 serotypes), microscopic agglutination test	Acute phase: negative, convalescent phase: negative
Viral investigation	
Hepatitis A (HA)	IgM-HA antibody: negative
Hepatitis B (HB)	HB surface antigen: negative, HB surface antibody: negative, HB core antibody: negative, HB e antigen: negative, HB e antibody: negative
Hepatitis C (HCV)	HCV antibody: negative, HCV core antigen: negative
Hepatitis E (HEV)	IgA-HEV antibody: negative
Epstein-Barr virus	VCA-IgG: 1:40, VCA-IgM: negative, EA-IgG: negative, EBNA: 1:20
Cytomegalovirus	Serological test: IgG (+), IgM (-)
Autoimmunological investigation	
Serum immunoglobulin level	IgG, IgG4, IgM: all within normal limit
Antinuclear antibodies	Negative
Anti-GBM antibody	Negative
PR-3 ANCA	Negative
MPO-ANCA	Negative
Anti-mitochondria antibody	Negative

On day seven of hospitalization, the patient recalled taking an OTC flu medication, Maoto and Kaigen® (Kaigen Pharma Co., Ltd., Osaka, Japan), approximately five days prior to hospital admission. Maoto is a Japanese herbal medicine composed of Ephedra herb, apricot kernel, cinnamon bark, and Glycyrrhiza root. Kaigen is a combination of herbs (Glycyrrhiza root, cinnamon bark, and ginger rhizome), acetaminophen, dl-methylephedrine hydrochloride, and caffeine anhydrous. We suspected DILI due to Maoto or Kaigen and subsequently performed a drug-induced lymphocyte stimulation test (DLST), which was positive for Kaigen. DLSTs for acetaminophen, dl-methylephedrine hydrochloride, and anhydrous caffeine were all negative. The Digestive Disease Week Japan (DDW-J) 2004 score was calculated as six points, indicating a high likelihood of DILI due to Kaigen [4].

**Figure 2:**
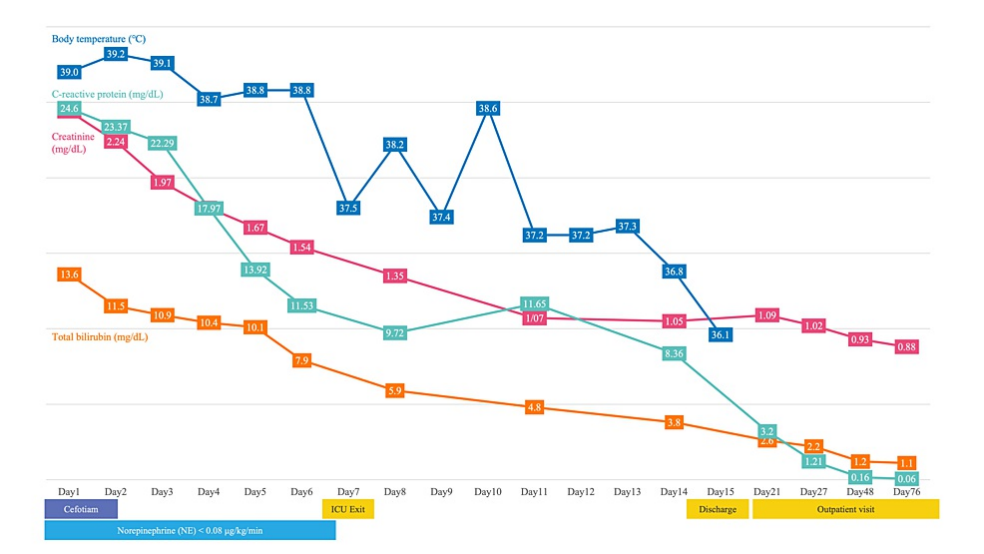
Overview of the clinical course The clinical course of the patient. Both the laboratory findings and the patient’s fever improved spontaneously.

As other causes were unlikely, the patient was diagnosed with DILI. We observed the clinical course of the patient, and both his laboratory data and fever improved spontaneously (Figure 2). The patient's condition was stable; therefore, he was discharged on day 15 of hospitalization. At the outpatient follow-up visit 61 days after discharge, the patient's liver injury, kidney injury, and inflammatory findings had all normalized.

## Discussion

In this case, we learned the following lessons when investigating liver injury. Firstly, liver injury can be severe even with OTC drugs. Secondly, while the DLST is available for investigating DILI, false positives, especially for medicinal herbs, should be noted, and it is necessary to adequately rule out other diseases. Finally, when the cause of liver injury is unclear, patient history taking should be repeated carefully.

In previous reports, DILI is most often caused by conventional medical care in the United States (US) and Europe, while traditional medical preparations caused DILI most often in Asia [1,5]. In this patient, the OTC herbal medication “Kaigen” was suspected to be responsible for DILI. Traditional Japanese herbal medicines, commonly referred to as "Kampo," are widely available in Japan and follow the same legal, regulatory, and logistical schemes as other conventional Western medicines. Some Kampo requires a doctor's prescription, while others, such as Kaigen, may be purchased by patients at their own discretion at drugstores. It has been shown that approximately 10% of patients with drug-induced jaundice die or require liver transplantation [1]. This was a case of cholestatic DILI with jaundice, which was potentially life-threatening. Due to the accompanying renal impairment, this case was classified as severe according to both the US Drug-Induced Liver Injury Network criteria [6] and the International DILI Expert Working Group criteria [7]. Adverse events associated with herbal medications and OTC medicines are often underestimated; however, as in this case, clinicians should be vigilant. In the present case, DLST was positive for Japanese herbal medicine [8]. Generally, DLST is an in vitro method that is useful for diagnosing drug hypersensitivity. However, it should be noted that false positives for DLST in Japanese herbal medicine are frequent [8]. Therefore, it is essential to carefully rule out diseases that mimic DILI, especially when herbal medicines are involved. When determining whether the liver injury is drug-induced, it is important to consider whether medication exposure precedes liver injury onset, whether causes of liver disease are ruled out, whether liver injury improves upon discontinuation of the drug, and whether repeated exposure to the drug causes severe recurrence [9]. This case met the first three of these four important factors. Moreover, other causes of liver injury were excluded according to the DDW-J 2004 criteria [4], and other infections and autoimmune diseases that can cause SIRS and organ damage were carefully ruled out. The DDW-J 2004 score was developed by modifying the Roussel Uclaf Causality Assessment Method (RUCAM) scale [10], which is an international diagnostic criterion for DILI. The DDW-J 2004 score includes the DLST as an evaluation component. We concluded that the etiology of this case was non-infectious due to the lack of response to the antibiotics, which prompted their discontinuation. With time, the SIRS and organ damage resolved spontaneously. Therefore, the patient was considered negative for sepsis. The clinical courses are also useful for differential diagnosis. Nonspecific symptoms such as fatigue, weakness, anorexia, fever, chills, and abdominal pain should also be considered manifestations of DILI, and these symptoms seem to influence poor outcomes and make the differential diagnosis of sepsis challenging [1].

If the etiology of liver injury is unclear, repeated history-taking is of utmost importance, as there may be an undisclosed history of drug ingestion. Medical history is also significant for obtaining key details to diagnose DILI such as drug intake preceding liver injury, improvement of liver injury after drug cessation, and exclusion of other causes of liver injury [9]. Clinicians asking patients to summarize and prioritize their medical history can be difficult, patients often do not understand what is normal, and the important information they should be relaying [11]. Therefore, a single medical history interview is insufficient. In this case, we asked the patient and his family repeatedly about their recent dietary, outdoor activity, and medication history. One week after admission, the patient recalled having taken OTC medications prior to hospital admission. One of the clinical cognitive errors is "premature closure," with humans tending to stop examining problems after finding an appropriate conclusion [12]. In this case, the patient was brought to us with a diagnosis of organ damage due to sepsis; however, we resisted premature closure and continued the investigation. It is clinically useful to withhold the diagnosis and declare the case "NYD (not yet diagnosed).” With herbal and non-prescription medications especially, the patient may be unaware that they have ingested medications [5]. If there is a possibility that DILI is present but not clear, a patient history should be obtained, and without premature closure of other diagnoses.

## Conclusions

The organ damage caused by OTC drugs and herbs can be difficult to diagnose and is potentially fatal. The DLST is informative for herb-induced organ damage, but false positives should be noted. Repeated history-taking and exclusion of other diseases are crucial for definitive diagnosis. Furthermore, the appropriate recognition of organ damage due to non-prescription drugs requires careful attention to avoid the premature termination of differential diagnoses.
